# Efficacy of balance training for hip fracture patients: a meta-analysis of randomized controlled trials

**DOI:** 10.1186/s13018-019-1125-x

**Published:** 2019-03-20

**Authors:** Jia-qi Wu, Lin-bo Mao, Jian Wu

**Affiliations:** 1 Rehabilitation Department, Jingjiang People’s Hospital, Jingjiang, Taizhou, Jiangsu Province China; 2 Institute Office, Jingjiang People’s Hospital, No. 28, Zhongzhou road, Jingjiang, Taizhou, 214500 Jiangsu Province China

**Keywords:** Osteonecrosis of femoral head, Core decompression, Autologous bone, Marrow cells implantation, Meta-analysis

## Abstract

**Background:**

To investigate whether the clinical effects of balance training were improved in hip fracture patients.

**Methods:**

Electronic databases which included PubMed, Embase, Web of Science, and the Cochrane Library up to December 2018 were searched. High-quality randomized controlled trials (RCTs) and prospective clinical controlled studies were selected based on inclusion criteria. Stata 12.0 was used for the meta-analysis. Standard mean difference (SMD) with 95% confidence interval (CI) was used to assess the effects.

**Results:**

Finally, 9 studies with 872 patients (balance training = 445, control = 427) were included in our meta-analysis (published between 1997 and 2018). Compared with the control group, balance training group showed a significant increase in overall function (SMD = 0.59, 95% CI [0.25, 0.93], *P* = 0.001), gait speed (SMD = 0.63, 95% CI [0.19, 1.07], *P* = 0.005), lower limb strength (SMD = 0.73, 95% CI [0.50, 0.95], *P* = 0.000), activities of daily living (ADLs) (SMD = 0.97, 95% CI [0.61, 1.34], *P* = 0.000), performance task scores (SMD = 0.41, 95% CI [0.21, 0.61], *P* = 0.000), and health-related quality of life (HRQoL) scores (SMD = 0.32, 95% CI [0.16, 0.47], *P* = 0.000).

**Conclusions:**

Our meta-analysis revealed that the balance training group has improved overall physical functioning, gait, lower limb strength, performance task, and activity of daily living than the control group. More high-quality and large-scale RCTs are needed to identify the optimal regimen of balance training after hip fracture.

## Background

Hip fractures are a common problem among older adults and can have a devastating impact on the ability of older patients to remain independent [1, 2]. However, individuals following hip fractures experience greater postural sway, possibly due to reduced muscular strength and proprioception [3]. Such physical limitations could hinder daily living and increase the risks of falls in patients following hip fracture compared to their healthy, age-matched counterparts [4].

Report has shown that 2 years after a hip fracture, more than half of men and 39% of women are dead or living in a long-term care facility [5]. In addition, balance deficit was the major risk factor for falls [6]. There is a need to identify optimal strategies to improve functional outcomes for hip fracture patients [7].

Evidence suggests that rehabilitation plays a role in guaranteeing recovery and enhancing quality of life following hip fracture [8]. And balance training could prevent falls in elderly individuals [9]. However, the effects of balance training for clinical outcomes in hip fracture patients were unknown. Therefore, it is necessary to conduct a meta-analysis comparing balance training for hip fracture patients.

Thus, we undertook a meta-analysis to evaluate whether balance training is superior to placebo with respect to (1) overall function, (2) gait speed, (3) lower limb strength, (4) activities of daily living (ADLs), (5) performance task scores, and (6) health-related quality of life (HRQoL). We hypothesized that balance training in the balance training group results in more hip function and higher limb strength than in the control group.

## Materials and methods

The current meta-analysis was performed according to the recommendations of the Cochrane Handbook for Systematic Reviews of Interventions and was reported in compliance with the Preferred Reporting Items for Systematic Reviews and Meta-Analyses (PRISMA) statement guidelines [10].

### Search strategy

**Fig. 1:**
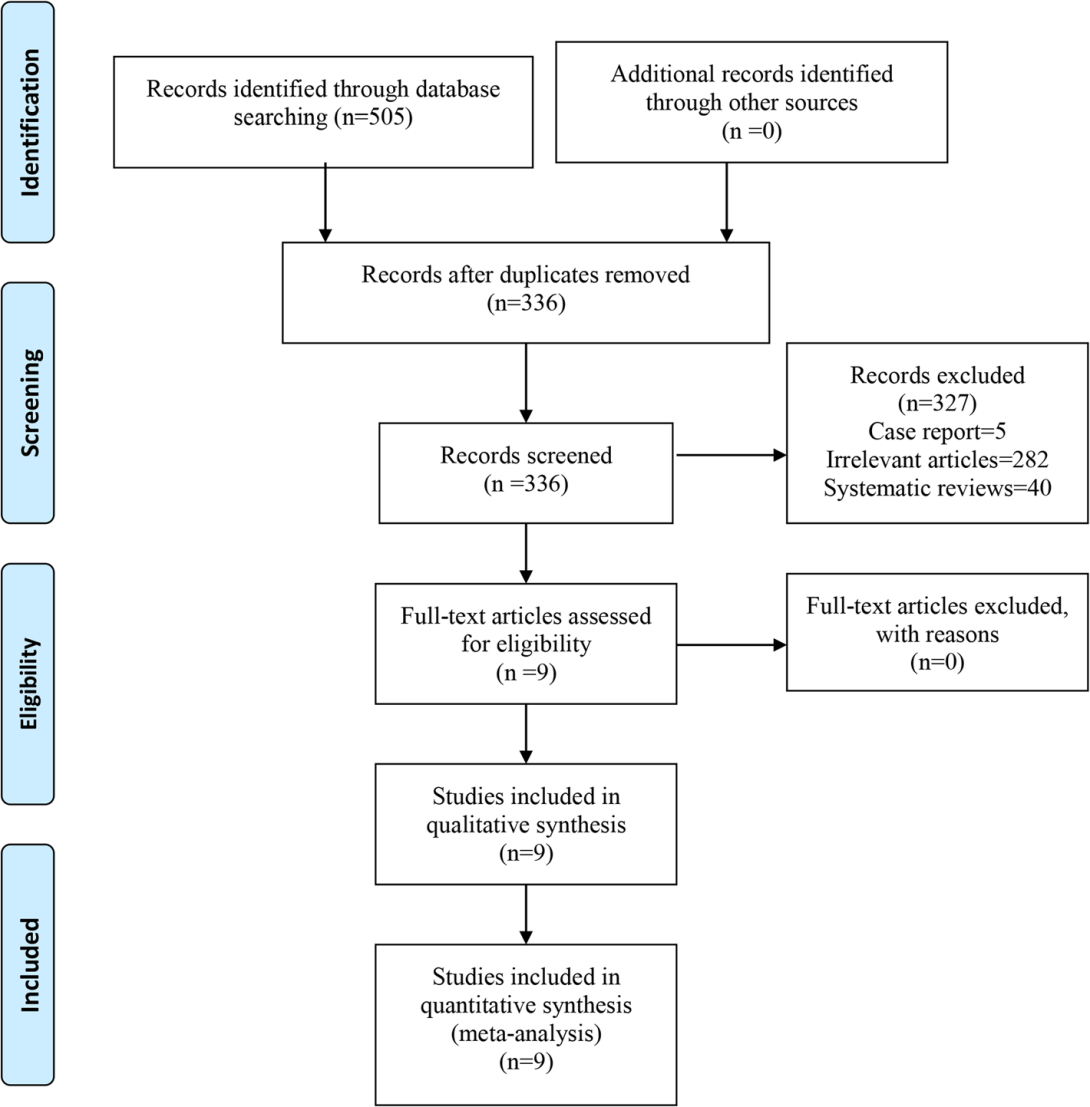
Flow diagram of the study selection process

Two reviewers performed an electronic literature search for randomized controlled trials (RCTs) or prospective clinical controlled studies comparing the balance training with control in the management of hip fracture. The electronic databases include PubMed, Embase, Web of Science, and the Cochrane Library up to December 2018. No language or date restrictions were applied. The following terms were used as keywords: ((((((((Fractures, Subtrochanteric) OR Subtrochanteric Fractures) OR Fractures, Intertrochanteric) OR Intertrochanteric Fractures) OR Fractures, Trochanteric) OR Trochanteric Fractures) OR Fractures, Hip)) AND (((((Training, Circuit) OR Circuit Training) OR Exercises, Circuit-Based) OR Exercise, Circuit-Based) OR balance training). In addition, further articles were obtained by reviewing references of the selected articles. The detail retrieval process is shown in Fig. 1.

### Inclusion criteria

Randomized controlled trials were included if they met the PICOS criteria as follows: Population: patients with hip fracture; Intervention: balance training; Comparator: placebo; Outcomes: overall function, gait speed, lower limb strength, ADLs, performance task scores, and HRQoL; Study design: RCTs or prospective clinical controlled studies.

### Data extraction

Two reviewers independently retrieved the relevant data from articles using a standard data extraction form. The extracted data included publication date, authors, study design, inclusion and exclusion criteria, number and demographics of participants, number of included hips, surgical procedure, duration of follow-up, and outcomes. For missing data, such as standard deviations, we tried to get it by contacting the original author first. If it did not work, we calculated missing standard deviations from other available data such as standard errors or the formulas in the Cochrane Handbook for Systematic Reviews of Interventions. Two reviewers extracted the data independently, and any disagreement was discussed until a consensus was reached.

### Risk of bias and quality assessment

The methodological bias and quality of included studies were assessed by The Cochrane Collaboration’s tool for assessing the risk of bias according to the Cochrane Handbook for Systematic Reviews of Interventions [11]. It is a two-part tool with seven specific domains: sequence generation, allocation concealment, blinding of participants and personnel, blinding of outcome assessment, incomplete outcome data, selective outcome reporting, and other sources of bias.

### Statistical and subgroup analysis

Stata 12.0 (Stata Corp., College Station, TX) was used to perform the meta-analysis. We used standard difference (SMD) and 95% confidence interval (CI) to assess continuous variable outcomes. For dichotomous outcomes, relative risks (RR) with a 95% CI were presented. Heterogeneity between studies was assessed by *I*^2^ and *χ*^2^ test. When *I*^2^ < 50% and *P* > 0.1, we used a fixed-effects model to evaluate; otherwise, a random-effects was used. In addition, subgroup analysis was performed to explore the source of heterogeneity when heterogeneity existed.

## Results

### Search results

The flowchart for the inclusion of articles is shown in Fig. 1. Initially, a total of 505 studies were searched via the databases and other sources (e.g., references). And 169 of the 505 studies were excluded due to the duplicates by Endnote Software (Version X7, Thompson Reuters, CA, USA). After reading the title and abstract, 327 trials were excluded according to the inclusion criteria. Finally, 9 studies [12–20] with 872 patients (balance training = 445, control = 427) were included in our meta-analysis (published between 1997 and 2018).

### General characteristic of the included studies

Table 1 shows the detailed characteristics of the trials included. Three studies were originated from the USA, three were from Australia, one from Germany, one from Italy and one from China. Sample size of the included studies ranged from 13 to 120. The type of balance training is different from each other. Duration ranged from 3 weeks to 6 months. Follow-up duration ranged from 1 to 12 months.

**Table 1:** General characteristic of the included RCTs

Author	Region	No. of patients (*n*)	Type of exercise	Control	Duration and frequency	Follow-up	Outcomes	Study
Binder 2004	USA	46/44	Phase 1: flexibility, balance, coordination, movement speed; phase 2: add progressive resistance training	Core exercise focused on flexibility	6 months (3 days a week)	6 months	3, 5, 6	RCT
Hauer 2002	Germany	15/13	Progressive functional training with walking, stepping, or balancing	Motor placebo activities (calisthenics games)	3 months (3 days a week)	3 months	1, 2, 3, 6, 7	RCT
Latham 2014	USA	120/112	Standing from a chair, climbing a step	Nutritional education	6 months (3 days a week)	9 months	1, 2, 4, 5, 7	RCT
Monticone 2018	Italy	26/26	Balance task-specific training while standing	Walking training and open kinetic chain exercise	3 weeks (3 days a week)	12 months	1, 2, 3, 4, 7	RCT
Moseley 2009	Australia	80/80	[Inpatient] Standing up, sitting down, tapping the foot, and stepping onto and off a block	Lower dose exercise (30 min/day)	4 months (14 days a week)	4 months	3, 4, 5	RCT
Peterson 2004	USA	38/32	With balance and gait training	Conventional physical therapy	2 months (2 days a week)	12 months	1, 2, 4, 6, 7	RCT
Sherrington 1997	Australia	20/20	Stepping exercise with weight-bearing exercise	No treatment	1 month (7 days a week)	1 month	1, 2, 4, 5, 7	RCT
Sherrington 2004	Australia	40/40	Sit-to-stand, lateral step-up, forward step-out-and-over, forward foot taps	Non-weight bearing exercise	4 months (NS)	4 months	1, 2, 3, 6, 7	RCT
Zheng 2010	China	60/60	With balance and gait training	No treatment	6 months (3 days a week)	6 months		RCT

*RCT* randomized controlled trials. 1, overall function; 2, gait; 3, lower limb strength; 4, ADLs; 5, performance task scores; 7, HRQoL

### Risk of bias in included studies

**Fig. 2:**
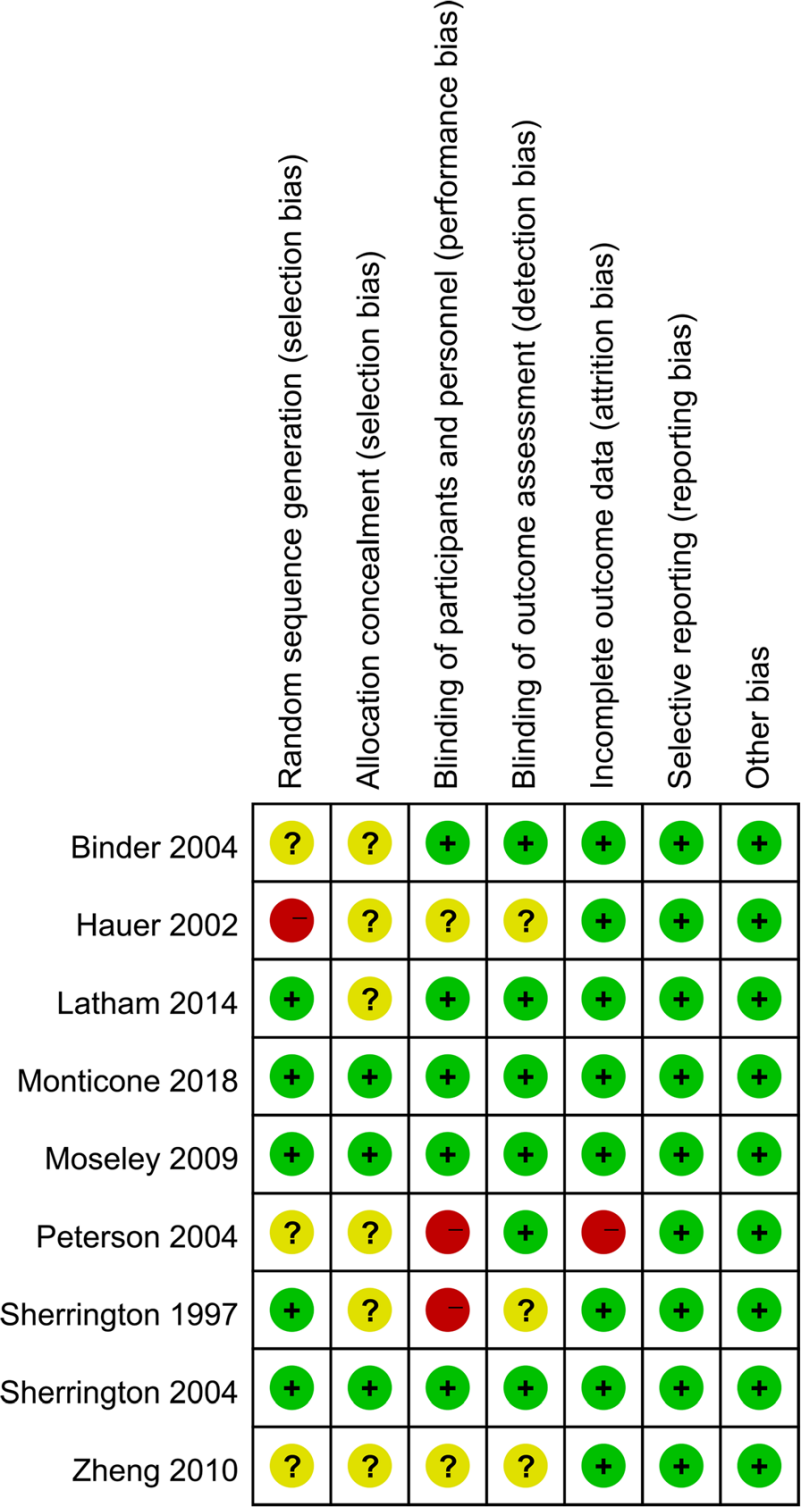
Risk of bias summary

**Fig. 3:**
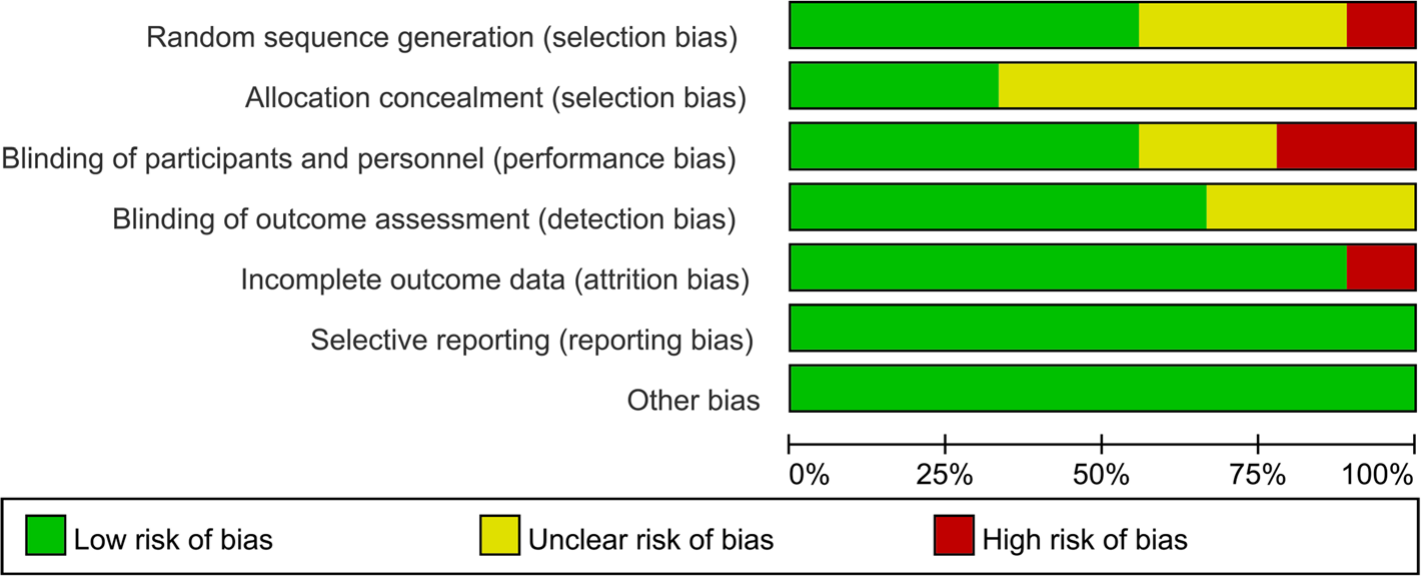
Risk of bias graph

Risk of bias summary and risk of bias graph are shown in Fig. 2 and Fig. 3 respectively. A total of three studies were rated as low risk of bias, three studies were qualified as high risk of bias, and the rest of the studies were rated as unclear risk of bias.

### Results of meta-analysis

#### Overall function

**Fig. 4:**
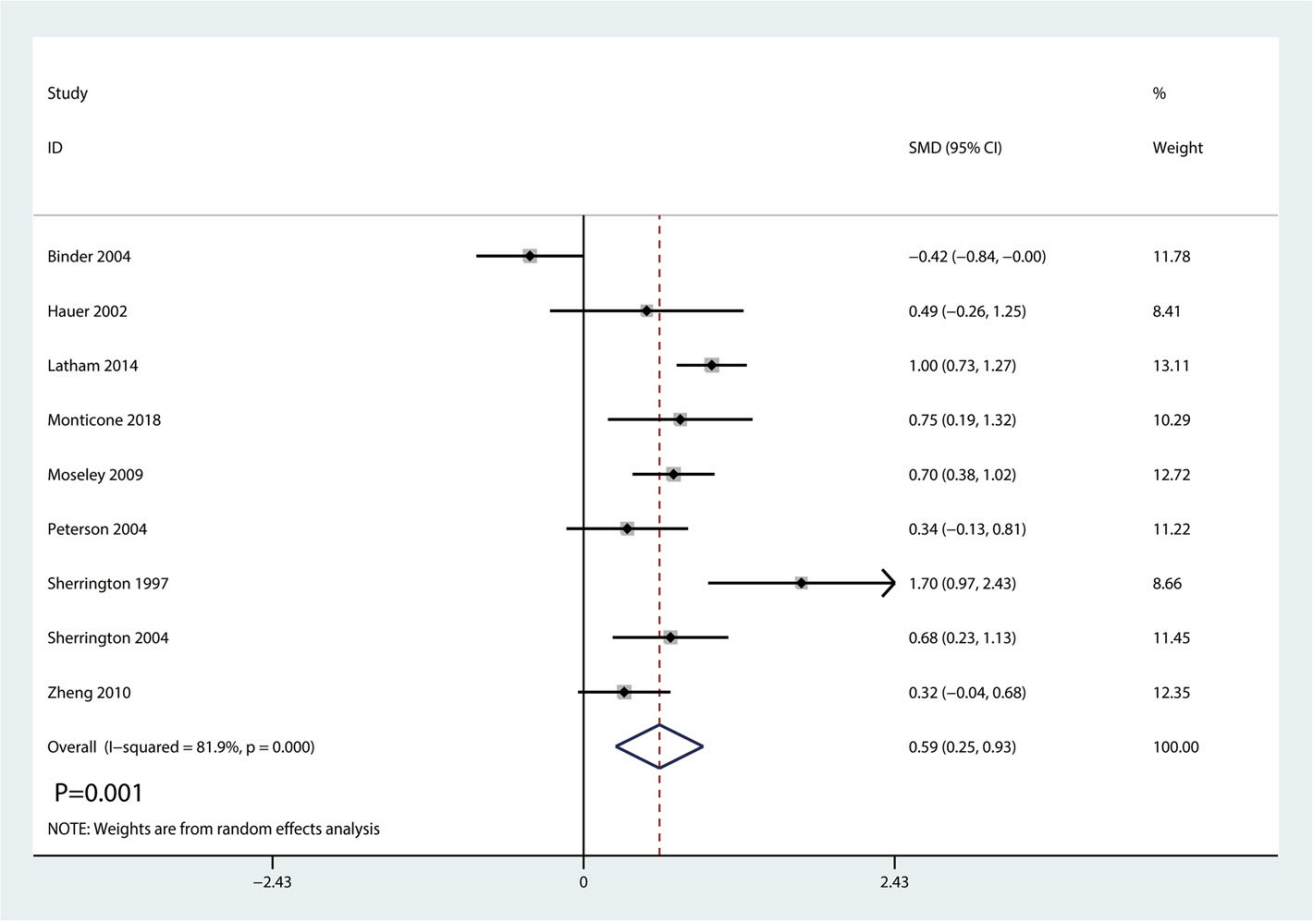
Forest plot for the comparison of overall function between the balance training group and control group

Nine studies enrolling 872 patients reported overall function postoperatively. There was a high heterogeneity existed between the nine studies (*I*^2^ = 81.9%; *P* = 0.000, Fig. 4). Thus, a random-effects model was performed. And there was a significant difference between the two groups (SMD = 0.59, 95% CI [0.25, 0.93], *P* = 0.001; Fig. 4).

#### Gait speed

**Fig. 5:**
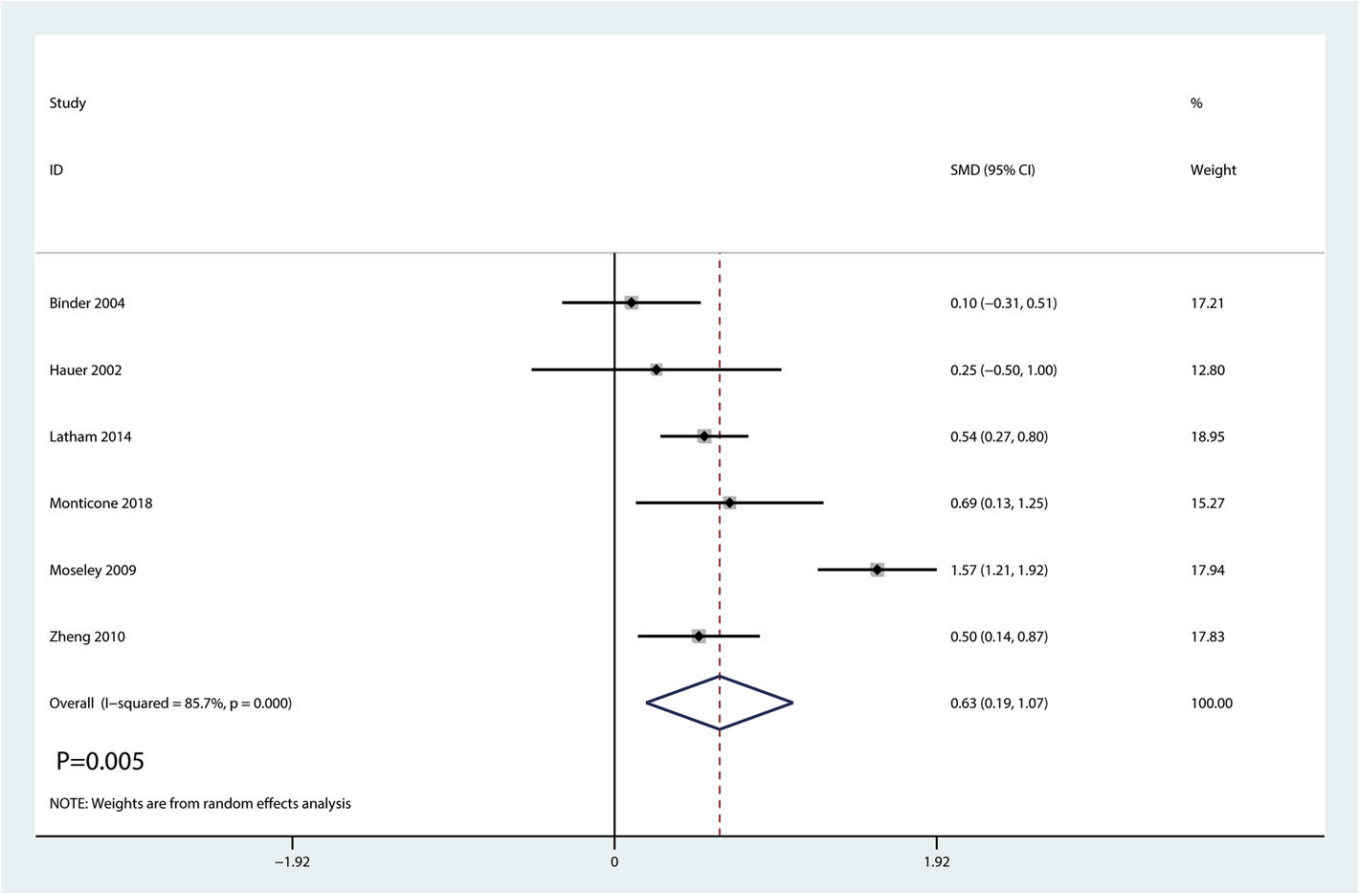
Forest plot for the comparison of gait speed between the balance training group and control group

Gait speed was reported in six studies enrolling 682 patients. Large heterogeneity existed between the six studies (*I*^2^ = 85.7%; *P* = 0.000, Fig. 5). So we adopted a random-effects model, and significant difference existed in the two groups (SMD = 0.63, 95% CI [0.19, 1.07], *P* = 0.005; Fig. 5).

#### Lower limb strength

**Fig. 6:**
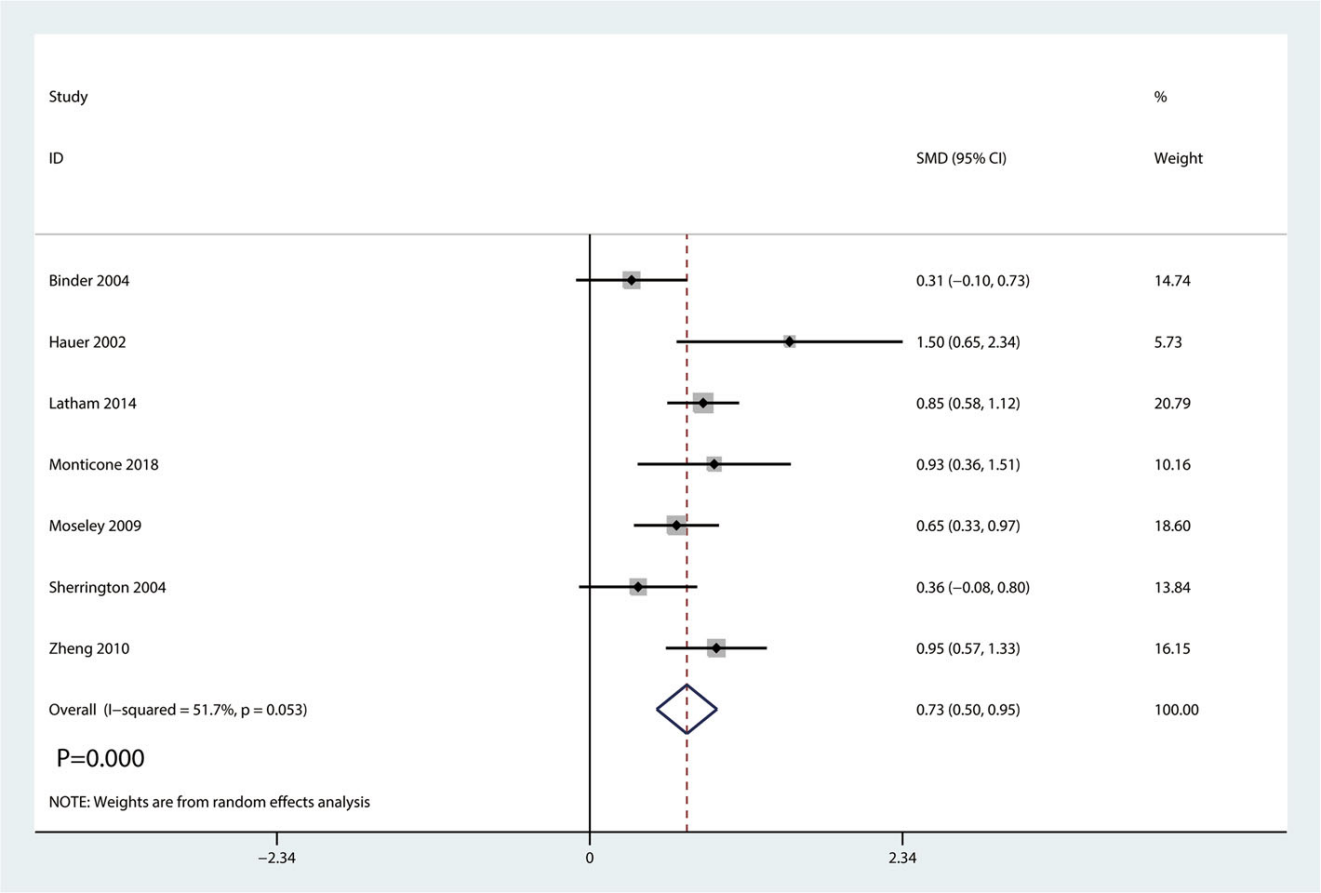
Forest plot for the lower limb strength between the balance training group and control group

Seven studies including 762 patients reported lower limb strength. Heterogeneity existed between the five studies (*I*^2^ = 51.7%; *P* = 0.053, Fig. 6). Thus, a random-effects model was performed. And meta-analysis showed balance training in the balance training group has a beneficial role in increasing lower limb strength than in the control group (SMD = 0.73, 95% CI [0.50, 0.95], *P* = 0.000; Fig. 6).

#### ADLs

**Fig. 7:**
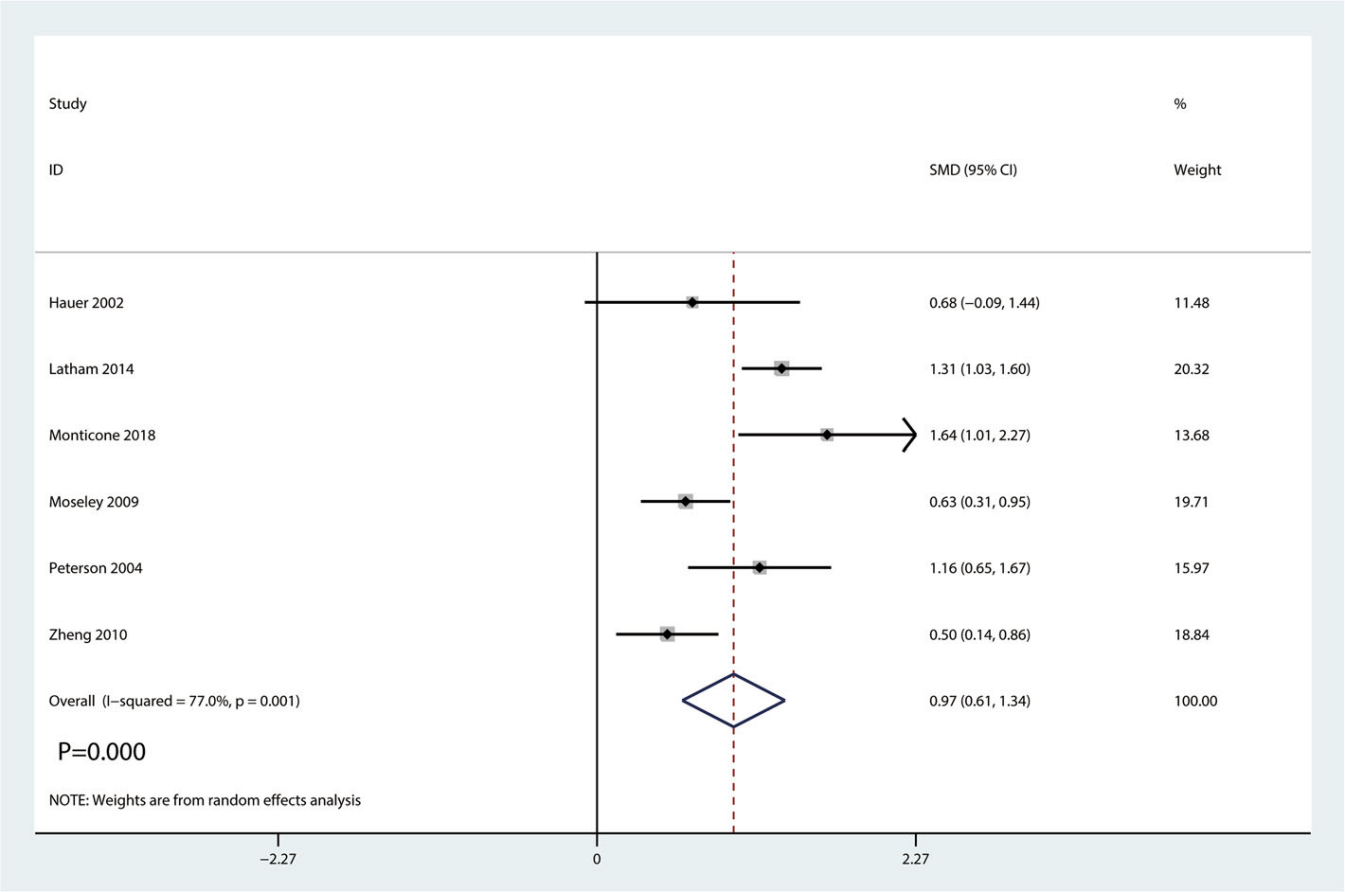
Forest plot for the comparison of ADLs between the balance training group and control group

Six studies including 662 patients reported the ADLs postoperatively. There was a high heterogeneity between the two studies (*I*^2^ = 77.0%; *P* = 0.001, Fig. 7). A random-effects model was conducted. And meta-analysis showed balance training in the balance training group has a beneficial role in increasing ADLs than in the control group (SMD = 0.97, 95% CI [0.61, 1.34], *P* = 0.000; Fig. 7).

#### Performance task scores

**Fig. 8:**
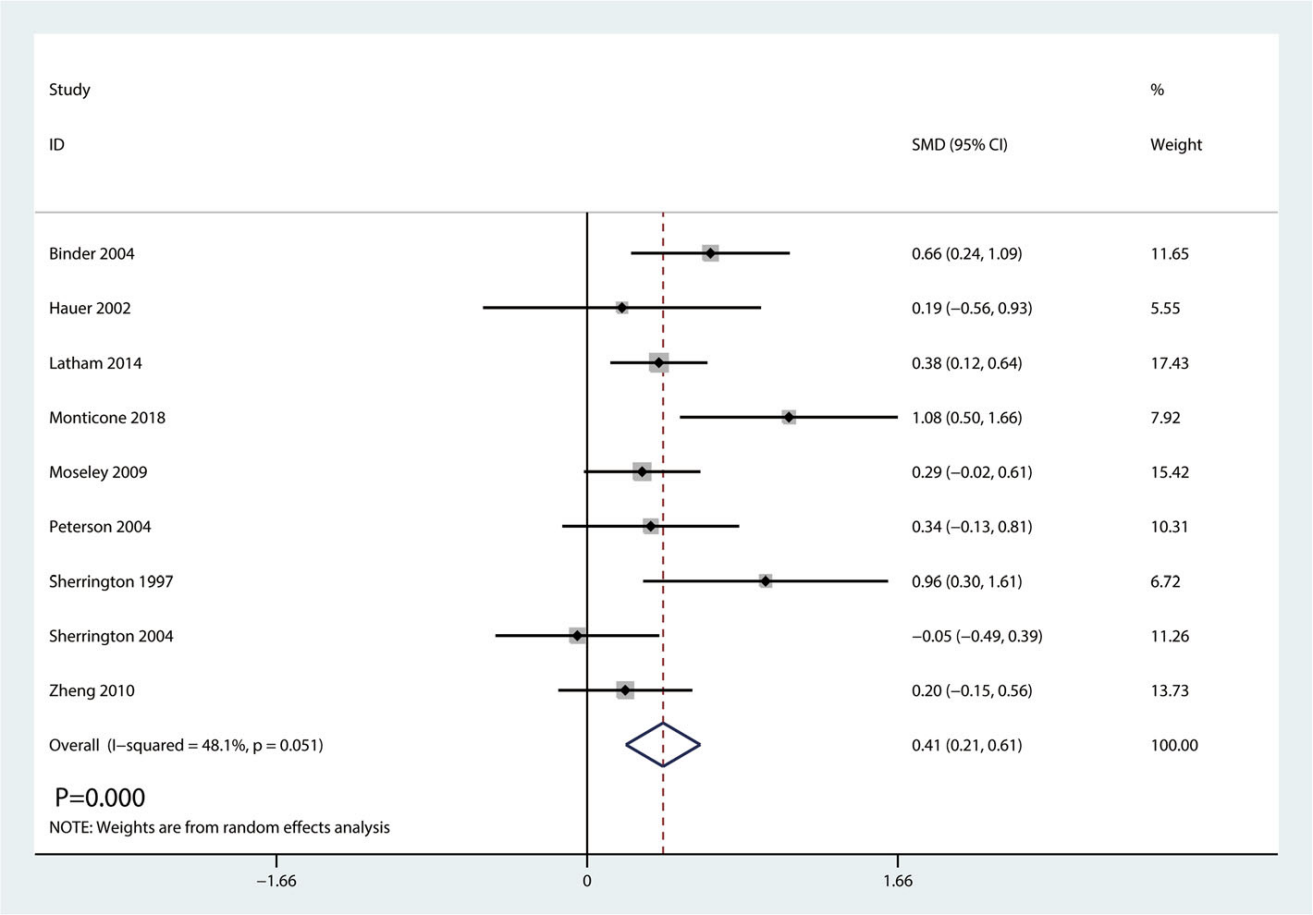
Forest plot for the comparison of performance task scores between the balance training group and control group

Nine studies including 872 patients reported performance task scores postoperatively. Mild heterogeneity existed between the three studies (*I*^2^ = 48.1%, *P* = 0.051, Fig. 8). So we conducted a random-effects model. Meta-analysis revealed that balance training could significantly increase the performance task scores (SMD = 0.41, 95% CI [0.21, 0.61], *P* = 0.000; Fig. 8).

#### HRQoL scores

**Fig. 9:**
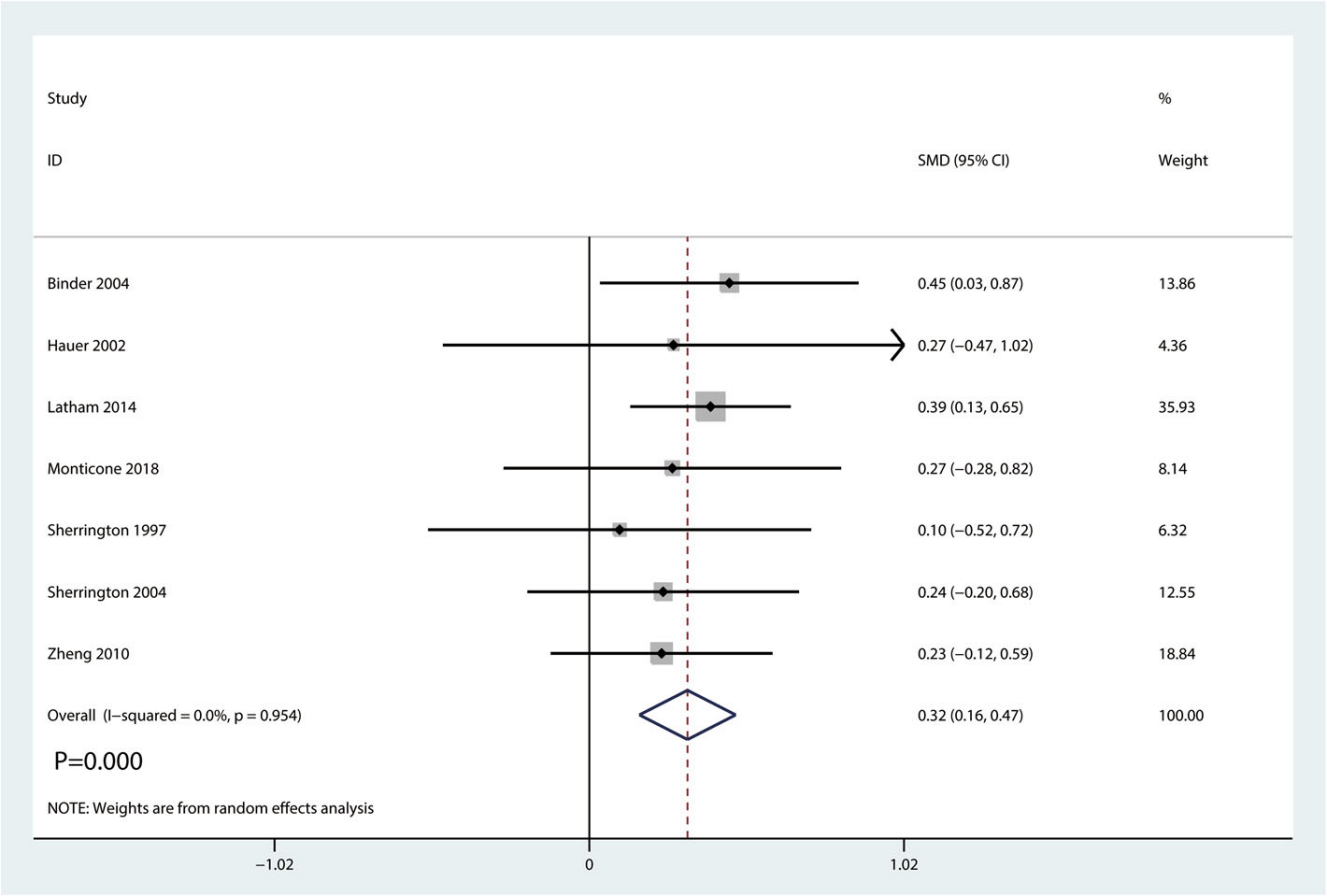
Forest plot for the comparison of HRQoL scores between the balance training group and control group

Seven studies including 642 patients reported HRQoL scores postoperatively. No heterogeneity existed between the seven studies (*I*^2^ = 0.0%, *P* = 0.954; Fig. 9). So we conducted a random-effects model. And meta-analysis showed significant difference between the two groups (SMD = 0.32, 95% CI [0.16, 0.47], *P* = 0.000; Fig. 9).

#### Publication bias, sensitivity analysis, and subgroup analysis

**Fig. 10:**
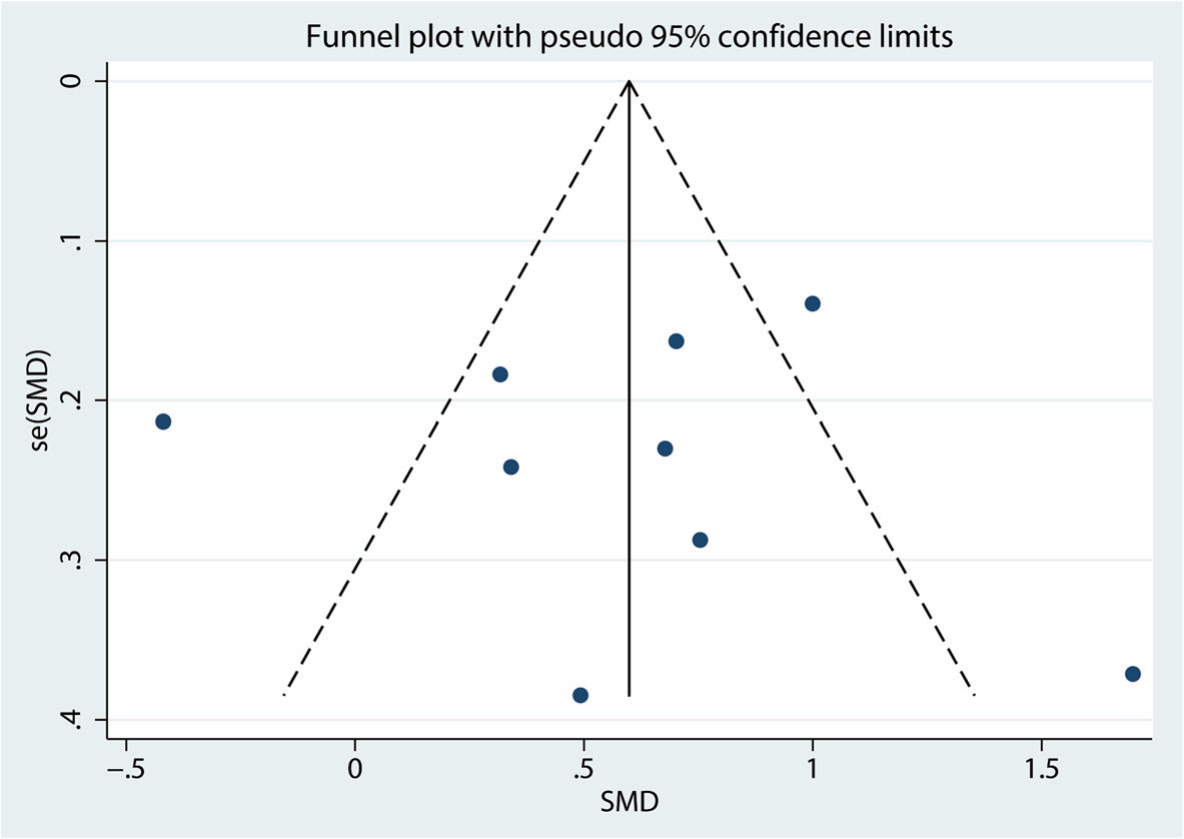
Funnel plot of the overall function between balance the training group and control group

**Fig. 11:**
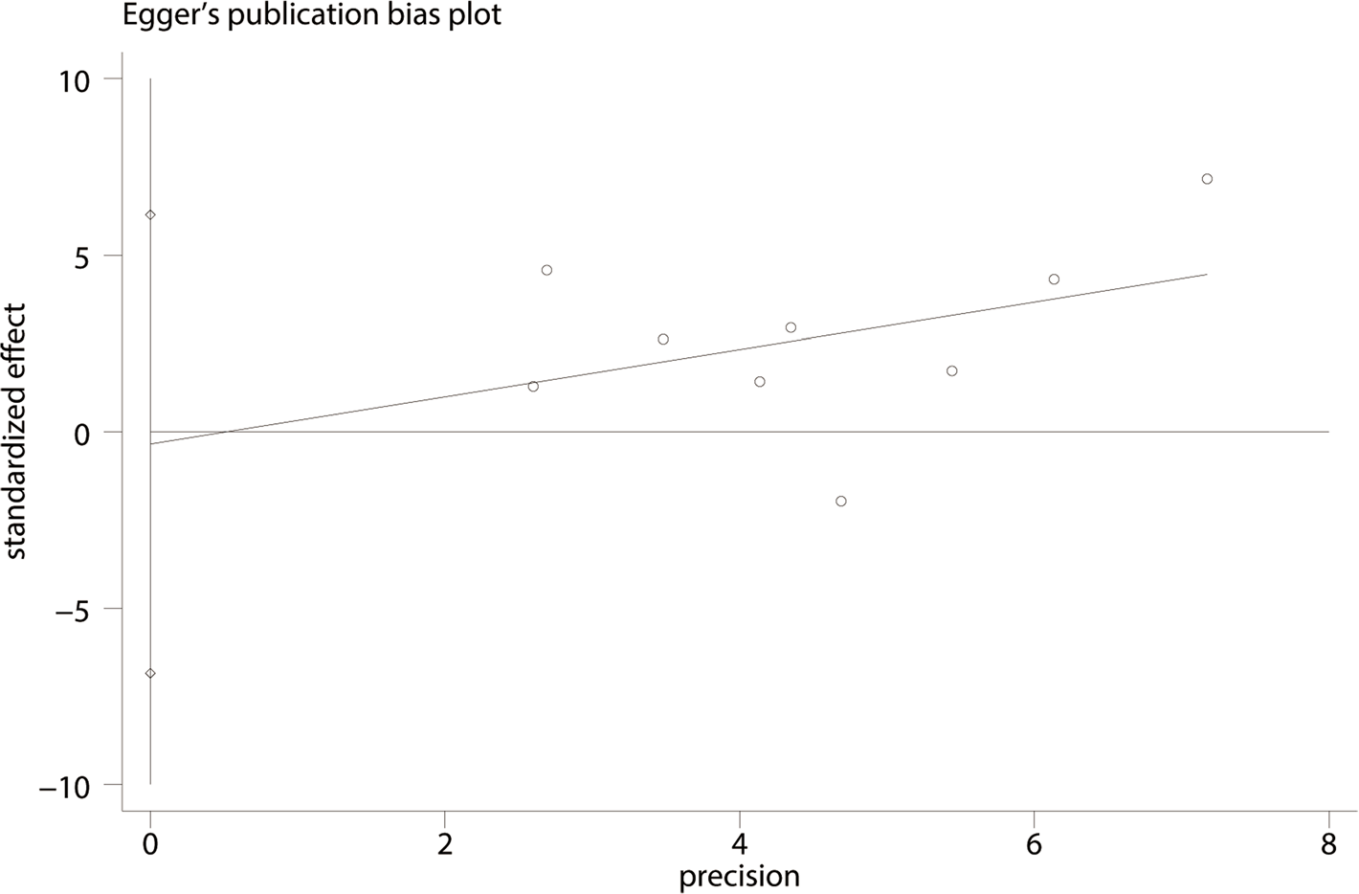
Egger’s test for overall function between the balance training group and control group

**Fig. 12:**
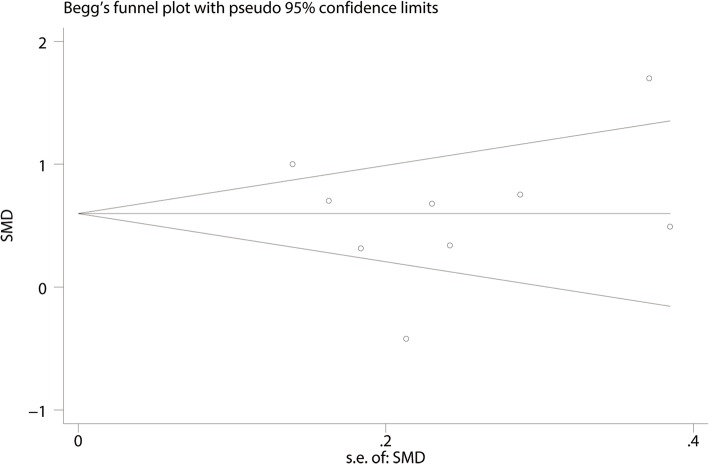
Begg’s test for overall function between the balance training group and control group

**Fig. 13:**
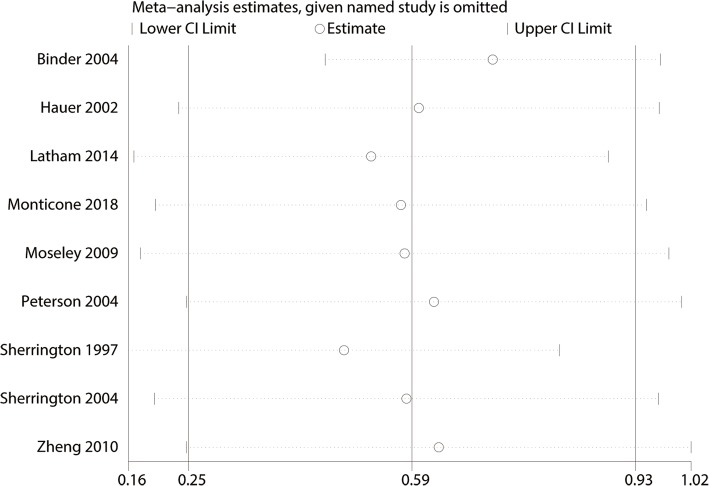
Sensitivity analysis for overall function between the balance training group and control group

For the meta-analysis of balance training on overall function, there was no evidence of publication bias by inspection of the funnel plot (Fig. 10) and formal statistical tests (Egger test, *P* = 0.69, Fig. 11; Begg test, *P* = 0.73, Fig. 12). Sensitivity analysis was performed by omitting included studies in turn, and results found that after removing each studies in turn, overall effect size was not changed (Fig. 13).

Subgroup analysis results are shown in Table 2. The findings of increased overall function were consistent in all subgroup analyses except for the frequency training and unclear/high risk of bias subgroups. Results found that high-frequency training is superior than low-frequency training in increasing overall function.

**Table 2:** Subgroup analysis results

Subgroup	No. of trials	Standard mean difference (95% CI)	*P* value	*I*^2^ (%)	Test of interaction, *P*
Total	9	0.59 (0.25, 0.93)	0.001	81.9	
Training duration					
≤ 3 months	3	0.71 (0.41, 1.01)	0.000	69.3	0.106
> 3 months	3	0.57 (0.41, 0.72)	0.000	88.2	
Risk of bias					
Low	3	0.70 (0.47, 0.94)	0.000	0.0	0.028
Unclear/high	3	0.54 (0.38, 0.71)	0.000	88.4	
Frequency of training					
≤ 3 days a week	7	0.52 (0.37, 0.68)	0.000	82.4	0.002
> 3 days a week	2	0.86 (0.57, 1.16)	0.000	83.5	
Follow-up					
< 6 months	5	0.78 (0.54,1.01)	0.000	57.8	0.105
≥ 6 months	1	0.50 (0.33,0.67)	0.000	88.1	

## Discussion

### Main findings

Results of this meta-analysis revealed that balance training has a positive role in increasing overall function for hip fracture patients. Moreover, balance training in the balance training group could significantly increase gait, lower limb strength, ADLs, performance task scores, and HRQoL scores than in the control group. Subgroup results have shown that high-frequency training was superior than low-frequency training for increasing overall function.

### Comparison with other meta-analyses

Only one relevant meta-analysis on the topic has been published [21]. Differences between ours and the previous ones should be noted. Different training frequency should be separately analyzed. Current meta-analysis performed publication bias, subgroup analysis, and subgroup analysis for overall function. Doma et al. [22] indicated that balance training improved walking capacity, balance-specific performance, and functional outcome measures for elderly individuals following total knee arthroplasty.

### Implications for clinical practice

Our meta-analysis showed that balance training could significantly increase overall function and lower limb strength after hip fracture. Moreover, high-frequency training was recommended. Latham et al. [14] revealed that the use of a home-based functionally oriented exercise program resulted in modest improvement in physical function at 6 months. However, the clinical importance of the balance training remains to be determined.

Previous studies have shown that people have poor functional outcomes after hip fractures [23]. Following fracture, patients are at a high risk of entering a vicious cycle like fear of falling as well as post-fracture pain and muscle weakness [24]. Previous exercise studies using intensive professional supervision and equipment have found a significant capacity for adults with hip fracture to improve after balance training [12, 25]. We included nine RCTs and found that balance training has a positive role in improving overall function, gait speed, and lower limb strength. ADLs were compared between the balance training and control group. Balance training is superior than the control group in terms of the ADLs. Moreover, balance training in the balance training group increased HRQoL scores than in the control group. Combs et al. [26] revealed that balance training could significantly increase health-related quality of life.

Overall, there were several strengths in our research which are as follows: (1) comprehensive retrieval strategy was applied to reduce the risk of publication bias, and (2) we performed sensitivity analysis and subgroup analysis to further increase the robustness of our final results.

Nevertheless, our meta-analysis does have certain limitations which needed to be addressed: (1) training frequency, duration, and follow-up were different, and thus, there is a large heterogeneity for the final outcomes; (2) follow-up duration was relatively short to assess the clinical effects of balance training; thus, long-term effects of balance training was needed; and (3) the detailed blind methods and allocation concealment were not described in some RCTs that may affect the validity of the overall findings.

## Conclusion

Our meta-analysis revealed that the balance training group has improved overall physical functioning, gait, lower limb strength, performance task, and activity of daily living than the control group. More high-quality and large-scale RCTs are needed to identify optimal regimen of balance training after hip fracture.

## Abbreviations

ADLs: Activities of daily living
CI: Confidence interval
HRQoL: Health-related quality of life
PRISMA: Preferred Reporting Items for Systematic Reviews and Meta-Analyses
RCTs: Randomized controlled trials
SMD: Standard mean difference
